# COVID-19 and obesity: a systematic review and meta-analysis on the pre-existing clinical conditions, COVID-19 symptoms, laboratory findings and clinical outcomes

**DOI:** 10.17179/excli2021-4226

**Published:** 2021-12-02

**Authors:** Maria Edilaine Rosário Ferreira, Arthur Vinícius Santos de Andrade, Alex André Ferreira Queiroz, Paulo Ricardo Martins-Filho, Eduardo Luis de Aquino Neves, Fernanda Oliveira de Carvalho, Adriano Antunes de Souza Araujo, Érika Ramos Silva, Paula Santos Nunes

**Affiliations:** 1 Health Sciences Graduate Program, Federal University of Sergipe, Aracaju, Sergipe, Brazil; 2 Tiradentes University, Aracaju, Sergipe, Brazil; 3 Professor Fernando Figueira Institute of Integral Medicine (IMIP), Recife, Pernambuco, Brazil; 4 Federal University of Sergipe, University Hospital (HU-UFS/EBSERH), Aracaju, Sergipe, Brazil

## ⁯

***Dear Editor,***

Coronavirus disease 2019 (COVID-19) is a primarily respiratory disease caused by the severe acute respiratory syndrome coronavirus 2 (SARS-CoV-2), a highly infectious RNA virus transmitted especially person-to-person by close contact through respiratory droplets. SARS-CoV-2 can infect cells with angiotensin-converting enzyme 2 (ACE2) receptor leading to a wide range of clinical manifestations including fever, fatigue, dry cough and dyspnea, gastrointestinal disorders, acute cardiac and renal injuries, and neurological manifestations (Hu et al., 2021[13]).

For some patients, the binding of the SARS-CoV-2 spike protein to the ACE2 may lead to a dysregulated inflammatory response and a massive release of pro-inflammatory cytokines implicated in an increased risk of multi-organ injury and death (Martins-Filho et al., 2020[17]). In addition, there is strong evidence that older age and pre-existing medical conditions including hypertension, diabetes, heart failure, and chronic obstructive pulmonary disease (COPD) are clinical risk factors for COVID-19 severity and mortality (Dessie and Zewotir, 2021[6]; Treskova-Schwarzbach et al., 2021[27]). However, contrasting results were found in relation to other comorbidities such as obesity. In a recent meta-analysis, it was shown that patients with obesity were at a higher risk of severe symptoms of COVID-19 rather than mortality (Geng et al., 2021[9]). In addition, it has been found that higher body mass index (BMI) is not related to a different immunological response in critically ill COVID-19 patients (Kooistra et al., 2021[15]). Here, we systematically review the literature on the clinical and laboratory characteristics, associated comorbidities, and outcomes of obese patients with COVID-19. 

This study followed the Preferred Reporting Items for Systematic Reviews and Meta-Analyses (PRISMA) guideline and was registered in the PROSPERO database (CRD42021228607). We performed a systematic search to identify relevant studies from PubMed, Web of Science, Scopus Embase, Google Scholar, bioRxiv and medRxiv. Searches were performed until April 1, 2021, with no language restriction, using the following terms: “COVID-19”, “SARS-CoV-2”, “severe acute respiratory syndrome coronavirus 2”, “2019-nCoV”, “coronavirus”, “coronaviruses”, “obesity”, “obesity, morbid” and “severe obesity”. We also conducted a hand search of cross-references from original articles and reviews to identify additional studies that could not be located in the electronic databases. 

Eligibility criteria were defined based on the PPO (population, predictors, and outcomes) elements: (1) population: patients with obesity (as defined by the study) aged 18 years or older and diagnosed with SARS-CoV-2 infection by real-time reverse transcription polymerase chain reaction (RT-PCR) or by serological testing; (2) predictors: pre-existing medical conditions, hematological and biochemical results, inflammatory markers, and coagulation function; (3) outcomes: the primary outcome was mortality and the secondary outcomes were admission to the intensive care unit (ICU) and orotracheal intubation. We included articles that presented information on pre-existing medical conditions, at least one laboratory parameter (white blood cells [WBC], neutrophils, lymphocytes, platelets, alanine transaminase [ALT], aspartate transaminase [AST], albumin, blood urea nitrogen, creatinine, lactate dehydrogenase [LDH], C-reactive protein [CRP], IL-6, serum ferritin, procalcitonin, and D-dimer) and mortality data. Two reviewers independently screened the search results and identified potentially relevant studies based on titles and abstracts. Relevant studies were read in full and included in the meta-analysis according to the eligibility criteria. 

The odds ratio (OR) with 95 % confidence interval (CI) was used as effect size for dichotomous variables (pre-existing medical conditions, symptoms, and outcomes of interest [death, ICU admission, and orotracheal intubation]). In addition, we calculated the standardized mean difference (SMD) for laboratory findings. Heterogeneity was quantified using the I^2^ index [100 % x (Q-df)/Q]. In the case of heterogeneity, we used the random-effects model to pool the results of individual studies, otherwise, the fixed-effects model was used. Forest plots were used to present the effect sizes and the 95 % CI, and 2-tailed p < 0.05 was used to determine significance. We conducted all analyses by using the Review Manager 5.4.1 (Cochrane IMS). 

The initial search found 12,561 references, of which 2334 were duplicates. After screening titles and abstracts, 112 full-text articles were assessed for eligibility and 99 were excluded. Finally, 13 studies (Al Heialy et al., 2021[1]; Biscarini et al., 2020[3]; Czernichow et al., 2020[5]; Hajifathalian et al., 2020[11]; Kang et al., 2020[14]; Kooistra et al., 2021[15]; McNeill et al., 2021[18]; Mehanna et al., 2021[19]; Moriconi et al., 2020[20]; Palaiodimos et al., 2020[22]; Petersen et al., 2020[23]; Smati et al., 2021[25]; Wolf et al., 2021[28]) were included in this systematic review (Supplementary Figure 1 in the supplementary file). Ten studies used a BMI ≥ 30 Kg/m² as a cut-off point for obesity and three studies used a BMI ≥ 25 Kg/m². A total of 9189 individuals were analyzed, 3421 obese (37.2 %) and 5768 (62.8 %) non-obese (Supplementary Table 1 in the supplementary file). Hypertension, diabetes, and pre-existing lung disease were more common in obese patients, as well as fever, dyspnea, and diarrhea as the first symptoms of COVID-19 (Supplementary Table 2 in the supplementary file). Increased levels of CRP (SMD = 0.55; 95 % CI 0.22 to 0.88), serum ferritin (SMD = 1.42; 95 % CI 0.61 to 2.22), and D-dimer (SMD = 1.10; 95 % CI 0.50 to 1.69) were found among obese patients (Supplementary Table 3 in the supplementary file). In addition, we found an association between obesity and ICU admission (OR = 1.52; 95 % CI 1.34 to 1.73) and orotracheal intubation (OR = 1.60; 95 % CI 1.37 to 1.86). There was no association between obesity and death (OR = 0.98; 95 % CI 0.75 to 1.28) (Supplementary Figure 2 in the supplementary file).

Obesity is a chronic disease associated with an increase in morbidity including hypertension, diabetes, and chronic kidney disease. Moreover, the impaired immunity in obese individuals has been considered a risk factor for severity in the presence of respiratory viral infections (Honce and Schultz-Cherry, 2019[12]). Excess adipose tissue may also favor the need for intubation in patients with respiratory infections due to biomechanical dysfunction with elastic impairment of the chest wall (pulmonary restriction and hypoventilation) and reduced airway caliber (Nakeshbandi et al., 2020[21]; Sattar et al., 2020[24]). 

There is evidence that obesity has a significant effect on lung function (Littleton, 2012[16]; Sattar et al., 2020[24]). Consistent with these findings, this study showed that obese patients with COVID-19 are more likely to have respiratory symptoms such as dyspnea compared to non-obese individuals. In addition, we found a higher frequency of fever and diarrhea as first symptoms of COVID-19 among obese patients. Our results are important because they indicate the need of monitoring and early management in obese patients presenting especially respiratory and gastrointestinal symptoms as the first symptoms of COVID-19. 

Despite the increased synthesis and release of pro-inflammatory cytokines in adipose tissue of the obese individuals (Coppack, 2001[4]) and the strong evidence of the association between “cytokine storm” and poor outcomes in patients with COVID-19 (Martins-Filho et al., 2020[17]), this study found no differences in the levels of IL-6 between obese and non-obese patients with SARS-CoV-2 infection. However, we found higher levels of CRP, serum ferritin, and D-dimer in obese patients, which indicates low-grade systemic inflammation and increased risk of hypercoagulation and thrombus formation (Eichinger, 2008[8]).

Interesting, the results of the present meta-analysis did not show an association between death and obesity for patients with COVID-19. This contradictory finding can be attributed to the “obesity paradox”, which postulates that obesity in older individuals or in patients with chronic clinical conditions can be protective and associated with decrease in mortality (Donini et al., 2020[7]). Despite the controversies regarding this “paradox” (Banack and Stokes, 2017[2]) and the limitations of crude anthropometric biomarkers such as BMI, it has been argued that nutritional status in obese elderly individuals and the health-deteriorating effect of undernutrition in non-overweight individuals probably contribute to this paradox (Hainer and Aldhoon-Hainerova, 2013[10]). However, we did not perform a meta-regression analyzing whether more severely obese individuals with COVID-19 are at high risk of death and a "U-shaped" outcome curve according to BMI should be further studied. Recently, a meta-analysis showed that association between BMI and obesity on composite poor outcomes (ICU admission, ARDS, severe COVID-19, use of mechanical ventilation, hospital admission, and mortality) was affected by age, gender, type 2 diabetes, and hypertension (Soeroto et al., 2020[26]).

In summary, this systematic review and meta-analysis showed that obese patients with COVID-19 are more likely to have hypertension, diabetes, and pre-existing lung disease; fever, dyspnea, and diarrhea as the first symptoms of SARS-CoV-2 infection; and higher levels of CRP, ferritin, and D-dimer than non-obese patients. Despite the association between obesity, ICU admission, and orotracheal intubation, no increased risk for mortality was found among obese patients with COVID-19. 

## Declaration

### Conflict of interest

There is no conflict of interest to declare. 

### Funding

There is no funding source. 

## Supplementary Material

Supplementary information
