# Supplementary material for: COVID-19 and obesity: a systematic review and meta-analysis on the pre-existing clinical conditions, COVID-19 symptoms, laboratory findings and clinical outcomes
Source: EXCLI J. 2021 Dec 2;20:1610–4. doi: 10.17179/excli2021-4226 (PMC8743833; doi:10.17179/excli2021-4226)
Supplement: Supplementary information [file EXCLI-20-1610-s-001.pdf]

EXCLI Journal 2021;20:1610-1614 – ISSN 1611-2156
Received: August 21, 2021, accepted: November 22, 2021, published: December 02, 2021

Supplementary information to:

Letter to the editor:

COVID-19 AND OBESITY: A SYSTEMATIC REVIEW AND META-ANALYSIS ON THE PRE-EXISTING CLINICAL CONDITIONS, COVID-19 SYMPTOMS, LABORATORY FINDINGS AND CLINICAL OUTCOMES

Maria Edilaine Rosário Ferreira[1*], Arthur Vinícius Santos de Andrade[3], Alex André Ferreira Queiroz[4], Paulo Ricardo Martins-Filho[1], Eduardo Luis de Aquino Neves[1], Fernanda Oliveira de Carvalho[1,2], Adriano Antunes de Souza Araujo[1], Érika Ramos Silva[1], Paula Santos Nunes[1]

1 Health Sciences Graduate Program, Federal University of Sergipe, Aracaju, Sergipe, Brazil
2 Federal University of Sergipe, University Hospital (HU-UFS/EBSERH), Aracaju, Sergipe, Brazil
3 Tiradentes University, Aracaju, Sergipe, Brazil
4 Professor Fernando Figueira Institute of Integral Medicine (IMIP), Recife, Pernambuco, Brazil

* **Corresponding author:** Maria Edilaine Rosário Ferreira, Health Sciences Graduate Program. Federal University of Sergipe, Rua Claudio Batista s/n, Aracaju, Sergipe, Brazil. CEP: 49060-100. E-mail: edilaine.rosario@hotmail.com

https://dx.doi.org/10.17179/excli2021-4226

This is an Open Access article distributed under the terms of the Creative Commons Attribution License (http://creativecommons.org/licenses/by/4.0/).

EXCLI Journal 2021;20:1610-1614 – ISSN 1611-2156
Received: August 21, 2021, accepted: November 22, 2021, published: December 02, 2021

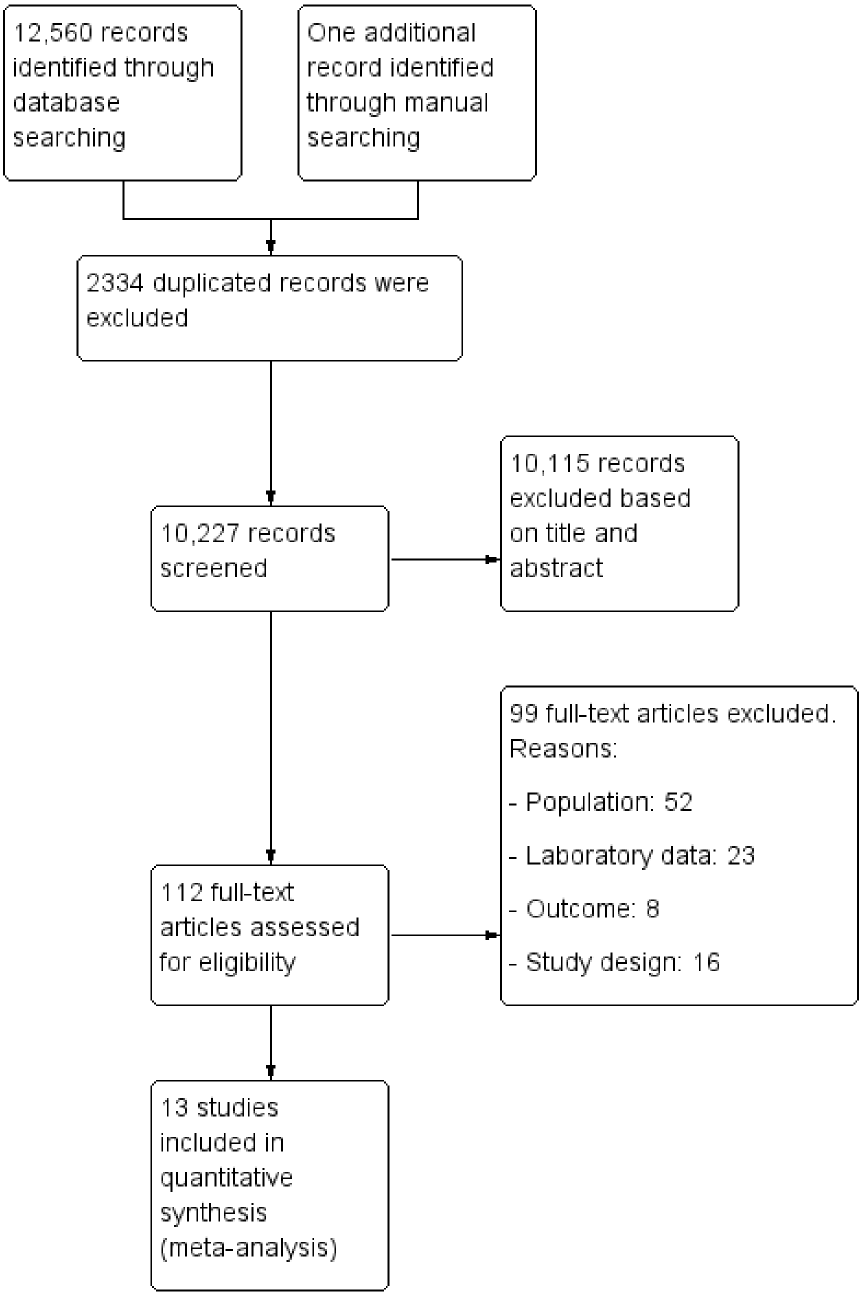

Supplementary Figure 1: Literature search flow diagram

EXCLI Journal 2021;20:1610-1614 – ISSN 1611-2156
Received: August 21, 2021, accepted: November 22, 2021, published: December 02, 2021

(A)

Study or Subgroup	Obese Events	Obese Total	Non-obese Events	Non-obese Total	Weight	Odds Ratio M-H, Random, 95% CI
Al Heialy et al. (United Arab Emirates - UAE)	15	102	15	184	2.7%	1.94 [0.91, 4.16]
Biscarini et al. (Italy)	26	80	48	252	4.9%	2.05 [1.16, 3.60]
Czernichow et al. (France)	591	1264	1102	2792	56.6%	1.35 [1.18, 1.54]
Hajifathalian et al. (USA)	92	277	104	493	13.3%	1.86 [1.34, 2.59]
McNeill et al. (USA)	129	349	113	432	15.4%	1.66 [1.22, 2.25]
Mehanna et al. (Egypt)	84	142	43	88	5.4%	1.52 [0.89, 2.59]
Palaiodimos et al. (USA)	29	162	3	38	1.0%	2.54 [0.73, 8.84]
Petersen et al. (Germany)	10	19	3	11	0.6%	2.96 [0.60, 14.73]
**Total (95% CI)**		**2395**		**4290**	**100.0%**	**1.52 [1.34, 1.73]**
Total events	976		1431			

Heterogeneity: Tau² = 0.00; Chi² = 7.47, df = 7 (P = 0.38); I² = 6%
Test for overall effect: Z = 6.53 (P < 0.00001)

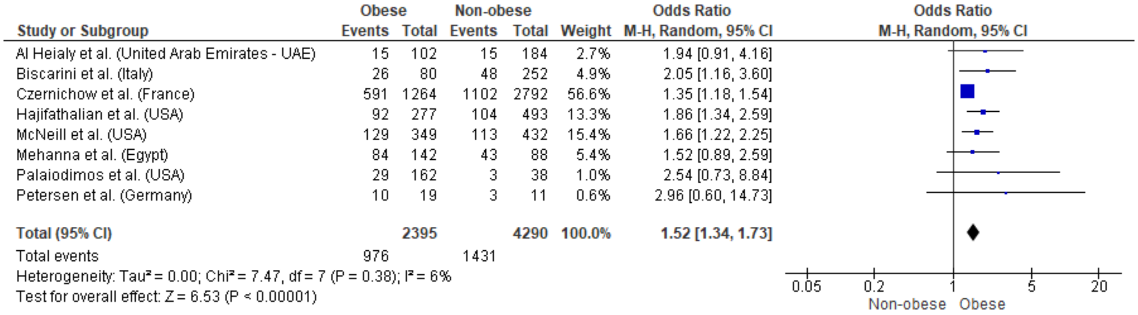

(B)

Study or Subgroup	Obese Events	Obese Total	Non-obese Events	Non-obese Total	Weight	Odds Ratio M-H, Fixed, 95% CI
Hajifathalian et al. (USA)	91	277	102	493	18.7%	1.88 [1.35, 2.61]
McNeill et al. (USA)	109	349	95	432	22.1%	1.61 [1.17, 2.22]
Palaiodimos et al. (USA)	35	162	7	38	3.4%	1.22 [0.50, 3.01]
Petersen et al. (Germany)	5	19	2	11	0.7%	1.61 [0.25, 10.13]
Smati et al. (France)	195	805	190	1159	44.7%	1.63 [1.30, 2.04]
Wolf et al. (USA)	40	138	39	139	10.5%	1.05 [0.62, 1.76]
**Total (95% CI)**		**1750**		**2272**	**100.0%**	**1.60 [1.37, 1.86]**
Total events	475		435			

Heterogeneity: Chi² = 3.80, df = 5 (P = 0.58); I² = 0%
Test for overall effect: Z = 6.07 (P < 0.00001)

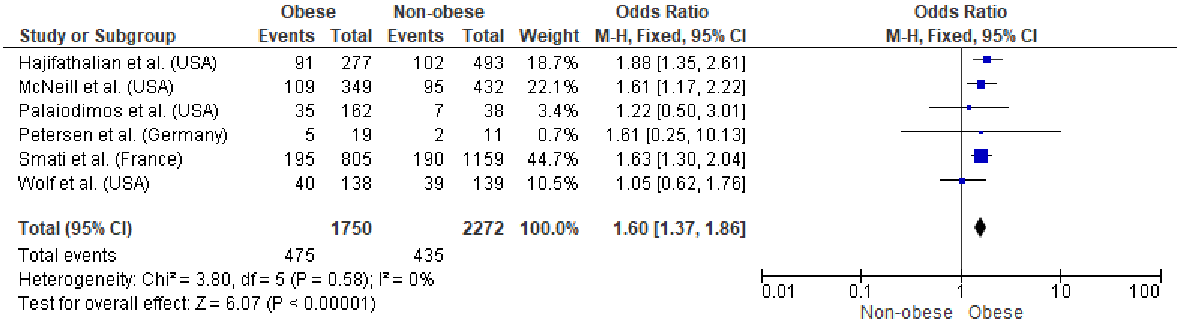

(C)

Study or Subgroup	Obese Events	Obese Total	Non-obese Events	Non-obese Total	Weight	Odds Ratio M-H, Random, 95% CI
Al Heialy et al. (United Arab Emirates - UAE)	6	102	4	184	3.5%	2.81 [0.77, 10.21]
Biscarini et al. (Italy)	26	80	89	252	10.5%	0.88 [0.52, 1.50]
Czernichow et al. (France)	244	1264	421	2792	16.5%	1.35 [1.13, 1.60]
Hajifathalian et al. (USA)	22	277	66	493	10.9%	0.56 [0.34, 0.93]
Kang et al. (China)	2	36	0	59	0.7%	8.62 [0.40, 184.87]
Kooistra et al. (The Netherlands)	3	18	12	49	3.1%	0.62 [0.15, 2.50]
McNeill et al. (USA)	49	349	67	432	12.8%	0.89 [0.60, 1.33]
Mehanna et al. (Egypt)	26	142	5	88	5.2%	3.72 [1.37, 10.09]
Moriconi et al. (Italy)	5	29	13	71	4.3%	0.93 [0.30, 2.89]
Palaiodimos et al. (USA)	36	162	12	38	7.2%	0.62 [0.28, 1.35]
Petersen et al. (Germany)	0	19	2	11	0.7%	0.10 [0.00, 2.24]
Smati et al. (France)	78	804	112	1160	14.5%	1.01 [0.74, 1.36]
Wolf et al. (USA)	27	138	35	139	10.0%	0.72 [0.41, 1.28]
**Total (95% CI)**		**3420**		**5768**	**100.0%**	**0.98 [0.75, 1.28]**
Total events	524		838			

Heterogeneity: Tau² = 0.11; Chi² = 30.91, df = 12 (P = 0.002); I² = 61%
Test for overall effect: Z = 0.16 (P = 0.87)

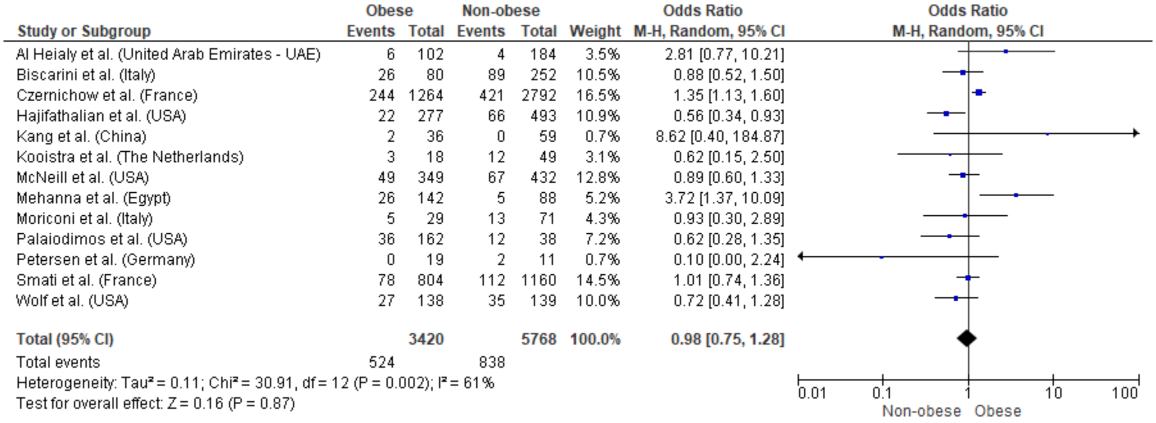

Supplementary Figure 2: Meta-analysis of association between obesity and ICU admission (**A**), orotracheal intubation (**B**), and death (**C**) for COVID-19 patients.

EXCLI Journal 2021;20:1610-1614 – ISSN 1611-2156
Received: August 21, 2021, accepted: November 22, 2021, published: December 02, 2021

Supplementary Table 1: Characteristics of the included studies

1st Author (country)	Year	Obesity	*n*	Male (%)	Age [a]	Non-obese (*n*)	Obese (*n*)	Outcome
Al Heialy et al. (UAE)	2021	BMI ≥ 30 kg/m^2	286	73%	46.9	184	102	ICU admission and death
Biscarini et al. (Italy)	2020	BMI ≥ 30 kg/m^2	332	69%	67	252	80	ICU admission and death
Czernichow et al. (France)	2020	BMI ≥ 30 kg/m^2	4056	66%	59.8	2792	1264	ICU admission and death
Hajifathalian et al. (USA)	2020	BMI ≥ 30 kg/m^2	770	60.8%	64	493	277	ICU admission, intu-bation, and death
Kang et al. (China)	2020	BMI ≥ 25 kg/m^2	95	41%	53	59	36	Death
Kooistra et al. (The Netherlands)	2021	BMI ≥ 30 kg/m^2	67	75%	NR	49	18	Death
McNeill et al. (USA)	2021	BMI ≥ 30 kg/m^2	781	58%	61	432	349	ICU admission, intu-bation, and death
Mehanna et al. (Egypt)	2021	BMI ≥ 30 kg/m^2	230	55%	46.5	88	142	ICU admission and death
Moriconi et al. (Italy)	2020	BMI ≥ 30 kg/m^2	100	52%	69.3	71	29	Death
Palaiodimos et al. (USA)	2020	BMI ≥ 25 kg/m^2	200	49%	64	38	162	ICU admission, intu-bation, and death
Petersen et al. (Germany)	2020	BMI ≥ 25 kg/m^2	30	60%	65.6	11	19	ICU admission, intu-bation, and death
Smati et al. (France)	2020	BMI ≥ 30 kg/m^2	1965	64.5%	70.1	1160	805	Intubation and death
Wolf et al. (USA)	2021	BMI ≥ 30 kg/m^2	277	63%	60	139	138	Intubation and death

BMI, Body mass index; *n,* Nº of patients; ICU, Intensive care unit; NR, not reported
[a] Mean or median

EXCLI Journal 2021;20:1610-1614 – ISSN 1611-2156
Received: August 21, 2021, accepted: November 22, 2021, published: December 02, 2021

Supplementary Table 2: Pre-existing medical conditions and symptoms among patients with COVID-19

Variables	Obese vs. non-obese	OR (95 % CI)	p-value	I^2
Pre-existing medical conditions				
Hypertension	65.2% vs. 57.7%	1.41 (1.17 – 1.70)	< 0.001	58%
Diabetes	58.0% vs. 50.2%	1.47 (1.32 – 1.63)	< 0.001	0%
Pulmonary disease	17.8% vs. 11.8%	1.57 (1.32 – 1.87)	< 0.001	0%
Heart disease	11.3% vs. 11.9%	0.84 (0.66 – 1.08)	0.17	51%
Cerebrovascular disease	7.1% vs. 10.3%	0.41 (0.10 – 1.72)	0.22	28%
Neoplasia	8.2% vs. 13.9%	0.64 (0.47 – 0.87)	0.005	45%
Kidney disease	11.4% vs. 13.9%	0.69 (0.59 – 0.82)	< 0.001	0%
Liver disease	7.2% vs. 5.4%	1.24 (0.72 – 2.13)	0.44	59%
COVID-19 symptoms				
Fever	77.1% vs. 72.9%	1.24 (1.00 – 1.54)	0.05	24%
Cough	68.0% vs. 61.6%	1.16 (0.88 – 1.53)	0.29	56%
Dyspnea	66.5% vs. 52.0%	1.86 (1.27 – 2.72)	0.001	75%
Nausea/vomiting	13.0% vs. 5.7%	1.51 (0.76 – 3.01)	0.24	0%
Myalgia/fatigue	46.5% vs. 42.4%	1.38 (0.94 – 2.02)	0.11	73%
Diarrhea	20.5% vs. 7.4%	1.96 (1.03 – 3.73)	0.04	44%

OR, odds ratio; CI, confidence interval

EXCLI Journal 2021;20:1610-1614 – ISSN 1611-2156
Received: August 21, 2021, accepted: November 22, 2021, published: December 02, 2021

Supplementary Table 3: Comparison of laboratory findings between obese and non-obese patients with COVID-19

Parameter	SMD (95% CI) between obese and non-obese individuals	p-value	I2
**Hematological**			
**WBC**	-0.04 (-0.17 to 0.08)	0.51	51%
**Neutrophils**	0.13 (-0.12 to 0.38)	0.29	66%
**Lymphocytes**	0.05 (-0.07 to 0.18)	0.40	47%
**Platelet count**	-0.05 (-0.16 to 0.06)	0.39	37%
**Biochemical**			
**ALT**	0.14 (-0.06 to 0.35)	0.17	54%
**AST**	0.04 (-0.14 to 0.23)	0.66	41%
**Albumin**	0.11 (-0.09 to 0.31)	0.27	47%
**Blood urea nitrogen**	-0.03 (-0.27 to 0.21)	0.83	-
**Creatinine**	0.04 (-0.39 to 0.46)	0.87	80%
**LDH**	0.74 (-0.06 to 1.54)	0.07	98%
**Infection-related indices**			
**CRP**	0.55 (0.22 to 0.88)	< 0.01	98%
**IL-6**	0.17 (-0.15 to 0.49)	0.29	90%
**Serum ferritin**	1.42 (0.61 to 2.22)	< 0.01	99%
**Procalcitonin**	0.13 (-0.19 to 0.46)	0.43	87%
**Coagulation function**			
**D-dimer**	1.10 (0.50 to 1.69)	< 0.01	98%

SMD, standardized mean difference; CI, confidence interval; WBC, white blood cells; ALT, alanine transaminase; AST, aspartate transaminase; LDH, lactate dehydrogenase; CRP, C-reactive protein. Positive results for SMD indicate increased levels of laboratory parameters in obese patients with COVID-19.
